# IRAP Endosomes Control Phagosomal Maturation in Dendritic Cells

**DOI:** 10.3389/fcell.2020.585713

**Published:** 2020-12-11

**Authors:** Mirjana Weimershaus, François-Xavier Mauvais, Irini Evnouchidou, Myriam Lawand, Loredana Saveanu, Peter van Endert

**Affiliations:** ^1^Institut National de la Santé et de la Recherche Médicale, Unité 1151, Université de Paris, Centre National de la Recherche Scientifique, UMR 8253, Paris, France; ^2^Inovarion, Paris, France

**Keywords:** aminopeptidase, oxytocinase, phagosome maturation, GLUT4-storage vesicle, cross-presentation, ERGIC

## Abstract

Dendritic cells (DCs) contribute to the immune surveillance by sampling their environment through phagocytosis and endocytosis. We have previously reported that, rapidly following uptake of extracellular antigen into phagosomes or endosomes in DCs, a specialized population of storage endosomes marked by Rab14 and insulin-regulated aminopeptidase (IRAP) is recruited to the nascent antigen-containing compartment, thereby regulating its maturation and ultimately antigen cross-presentation to CD8^+^ T lymphocytes. Here, using IRAP^–/–^ DCs, we explored how IRAP modulates phagosome maturation dynamics and cross-presentation. We find that in the absence of IRAP, phagosomes acquire more rapidly late endosomal markers, are more degradative, and show increased microbicidal activity. We also report evidence for a role of vesicle trafficking from the endoplasmic reticulum (ER)–Golgi intermediate compartment to endosomes for the formation or stability of the IRAP compartment. Moreover, we dissect the dual role of IRAP as a trimming peptidase and a critical constituent of endosome stability. Experiments using a protease-dead IRAP mutant and pharmacological IRAP inhibition suggest that IRAP expression but not proteolytic activity is required for the formation of storage endosomes and for DC-typical phagosome maturation, whereas proteolysis is required for fully efficient cross-presentation. These findings identify IRAP as a key factor in cross-presentation, trimming peptides to fit the major histocompatibility complex class-I binding site while preventing their destruction through premature phagosome maturation.

## Introduction

Dendritic cells (DCs) are immune myeloid cells that exert prime functions in the initiation of adaptive immune responses. Key to the activation of cytotoxic CD8^+^ T lymphocytes, the effector cells required for immunity against most viruses and cancer, is the generation of stable peptide-major histocompatibility complex class (MHC)-I complexes (pMHC) on the surface of DCs (Jung et al., 2002). While all nucleated cells generate pMHC loaded with peptides from proteins encoded in the cellular genome in a process referred to as the classical pathway of MHC-I antigen presentation, few cell types, including DCs, are able to “cross-present” and thus activate CD8^+^ T lymphocytes with peptides derived from extracellular proteins. As the spatially restricted peptide-binding pocket on MHC-I molecules is best stabilized by 8-mer and 9-mer peptides, an appropriate hydrolytic machinery in antigen-presenting cells is required in order to break down complex protein antigens into fragments of a suitable size (reviewed in Rock et al., 2010). While the C-terminus of most MHC-I-associated peptides is generated by the proteasome, different N-terminal trimming peptidases have been identified (reviewed in Weimershaus et al., 2013). In this context, we have discovered an unexpected role as trimming enzyme for insulin-regulated aminopeptidase (IRAP), a widely expressed enzyme best studied for its role in oxytocin and vasopressin cleavage and its colocalization with glucose transporter (GLUT) 4 to specific cytoplasmic insulin-responsive storage vesicles (GSVs; reviewed in Li et al., 2019).

Insulin-regulated aminopeptidase is structurally and ontogenetically closely related to the endoplasmic reticulum (ER) aminopeptidases (ERAPs). However, while ERAPs are soluble enzymes in the ER lumen, IRAP is a type II transmembrane protein located in specific endosomes that in DCs contain also the small guanosine triphosphatase (GTPase) Rab14. Its trafficking is regulated by cell type-specific extracellular signals and requires the short cytoplasmic tail of IRAP (Kandror and Pilch, 2011). While adipocytes and muscle cells mobilize IRAP vesicles in response to insulin, we have shown that in DCs, ligation of Fc receptors (FcRs) by immune complexes triggers phosphorylation and inactivation of the Rab14 GTPase-activating protein (GAP) AS160/Tbc1d4, resulting in the activation of Rab14 (Weimershaus et al., 2018). Active Rab14-GTP can form a complex with the kinesin Kif16b, which enables plus-end-directed transport of the vesicles on microtubules and promotes fusion with early endosomes and phagosomes (Weimershaus et al., 2018).

Thus, IRAP vesicle trafficking intersects with endocytic and phagocytic pathways. Phagocytosis is an evolutionary ancient process by which cells internalize extracellular particles and include them in membrane-bound organelles in a receptor-dependent manner. While generally targeted for destruction upon fusion with highly degradative lysosomes (Fairn and Grinstein, 2012), phagosomes in DCs display particular features with regard to their fusion and fission dynamics as well as in the composition of their enzymatic arsenal. Thus, the DC phago-endosomal system seems to be optimized in order to preserve peptides of suitable size for loading on MHC-I molecules for cross-presentation (Amigorena and Savina, 2010). Several studies have added to our mechanistical understanding how DC phagosome maturation and therefore cross-presentation are regulated by different intracellular membrane-modifying and fusion components. Rab GTPases and soluble N-ethylmaleimide–sensitive factor attachment protein receptor (SNARE) tethers are master regulators of vesicular trafficking (Stenmark, 2009). Several factors have been reported to interfere with phagosome maturation and cross-presentation in DCs: Rab27a that brings NADPH oxidase 2 (NOX2)-containing vesicles to the phagosome (Jancic et al., 2007); Rab39a regulating ER–Golgi-to-phagosome transport and stabilizing phagosomal NOX2 and MHC-I molecules (Cruz et al., 2020); Rab3b/c (Zou et al., 2009), Rab11, and Rab22 involved in Toll-like receptor (TLR)-triggered MHC-I delivery to phagosome (Nair-Gupta et al., 2014); Rab34 inducing lysosomal clustering to the perinuclear region (Alloatti et al., 2015); and Rab43, a Golgi-related Rab controlling cross-presentation of phagocytized antigen *in vivo* (Kretzer et al., 2016). The SNARE protein Sec22b, by interaction with Stx4 on phagosomes, has been suggested to supply the latter with components of the peptide-loading complex and facilitate cross-presentation, although this role has been challenged by a second group using an *in vivo* model (Montealegre and van Endert, 2017; Wu et al., 2017).

A non-canonical role of proteins involved in autophagy also affects phagosome maturation, although the regulation of this pathway is incompletely understood. In this process, upon TLR or apoptotic cell receptor engagement, several components classically involved in macroautophagy sequentially associate with phagosomes and modulate their maturation. This so-called LC3-associated phagocytosis (LAP) is characterized by sustained recruitment of NOX2 and accelerated fusion with lysosomes leading to more efficient degradation of phagocytized substrates in murine macrophages and enhanced presentation of phagocytized antigens by MHC class II molecules (Sanjuan et al., 2007; Heckmann and Green, 2019).

While detrimental for cross-presentation when accelerated, phago-lysosomal fusion is the default end point of phagocytosis and required for peptide presentation on MHC-II and the destruction of internalized pathogens. The requirement to balance these functions against efficient cross-presentation underlines the importance for DCs of fine-tuning the endo-lysosomal system according to different cellular functions and extracellular cues (Samie and Cresswell, 2015).

Here we investigate how phagosome maturation specifically in DCs is controlled by fusion with an endosomal compartment marked by IRAP and Rab14, suggest that vesicle transport from the ER–Golgi intermediate compartment (ERGIC) affects IRAP vesicles, and confirm the role of IRAP peptidase activity in cross-presentation.

## Materials and Methods

### Mice and Cells

Previously described IRAP^–/–^ mice on an Sv129 background obtained from S. Keller were back-crossed up to 10 times to C57BL/6 mice obtained from Janvier (St. Quentin-Fallavier, France). While for some experiments mice with a lower number of back-crosses were used, cross-presentation experiments were always performed with mice on an identical genetic background. Control mice were either mixed background or C57BL/6 mice bred in our facility or C57BL/6 mice purchased from Janvier. Recombination-activating gene (RAG)1-deficient OT-1 T cell receptor transgenic mice were obtained from Taconic (Germantown, NY, United States) and bred in our animal facility. Animal experimentation was approved by the *Comité d’Éthique pour l’Expérimentation Animale* Paris Descartes (no P2.LS.156.10). Murine bone marrow-derived DCs (BM-DCs) were produced *in vitro* by culturing cells extruded from large bones for 6–8 days in complete medium [Iscove’s modified Dulbecco’s medium (IMDM) complemented with 10% fetal calf serum (FCS), 2 mM glutamine, 100 U/ml penicillin, 100 g/ml streptomycin, 50 mM β-mercaptoethanol supplemented with J558 supernatant containing 20 μg/ml granulocyte-macrophage colony-stimulating factor (GM-CSF)]. Bone marrow-derived DC differentiation and activation were checked by staining with CD11c and CD80 antibodies as described (Weimershaus and van Endert, 2013).

### Antibodies

The following antibodies were used in this study.

#### Fluorescence Microscopy

immunoaffinity purified rabbit antibodies specific for the cytosolic IRAP domain (Keller et al., 1995); mouse monoclonal IRAP antibodies (a kind gift from M. Birnbaum, University of Pennsylvania) (Garza and Birnbaum, 2000); goat polyclonal anti-EEA1 and anti-mouse transporter 1, ATP-binding cassette subfamily B member transporter associated with antigen processing (TAP1) (both Santa Cruz Biotechnologies); rat anti-mouse lysosome-associated membrane protein (LAMP)1 clone 1D4B, mouse monoclonal anti-STX6, mouse monoclonal anti-GM130 (BD Pharmingen); rabbit polyclonal anti-STX6 (ProteinTech Group, Chicago, IL, United States); rat monoclonal anti-mouse mannose receptor, clone MR5D3 (AbD Serotec); rabbit polyclonal anti-Sec22b (Synaptic Systems); rabbit polyclonal anti-Rab14 (Sigma Aldrich); rat monoclonal anti-HA tag (Roche); rabbit polyclonal anti-LC3 (MBL). All secondary reagents were Alexa-coupled highly cross-adsorbed antibodies from Molecular Probes (Invitrogen).

#### Immunoblotting

In addition to antibodies also used for microscopy, the following antibodies were used for immunoblots: rabbit polyclonal anti-MHC-I (P8, a gift from H. Ploegh); rabbit polyclonal anti-EEA1 (Abcam); rabbit polyclonal anti-TAP2 (a gift from Dr. J. Monaco); rabbit polyclonal anti-calnexin (Stressgen); rabbit polyclonal anti-V-ATPase subunit E and goat polyclonal anti-Cathepsin D (both Santa Cruz Biotechnologies).

#### PhagoFACS Assays

PhagoFACS assays: (additional antibodies) rabbit polyclonal anti-ovalbumin (OVA) (Sigma Aldrich); mouse monoclonal anti-Rab7 (Abcam); rabbit polyclonal anti-BSA (Invitrogen).

#### Flow Cytometry and Sorting

Flow cytometry and sorting: (additional antibodies) rat anti-mouse CD11b/PE-Cy7 (clone M1/70; BD Biosciences); hamster anti-mouse CD11c/eFluor450 (clone N418; eBioscience); rat anti-H2-K^b^ (clone AF6-88.5; Biolegend); mouse IgG2a isotype control (clone MOPC-173, Biolegend); 7-actinomycin D (7-AAD, BD Biosciences).

#### ELISAs

Capture: rat anti-mouse interleukin (IL)-2 (clone JES6-1A12); detection: rat anti-mouse IL-2/Biotin (clone JES6-5H4; both BD Biosciences).

### Fluorescence Microscopy

Bone marrow-derived DCs on day 7 were plated on fibronectin-coated 12-mm coverslips for 3 h in complete medium at 37°C. The cells were fixed in 4% paraformaldehyde (PFA) for 20 min at room temperature (RT), permeabilized in 0.2% saponin/0.2% bovine serum albumin (BSA) in phosphate buffered saline (PBS), quenched in 0.2 M glycine pH 7 and incubated with antibodies. For phagocytosis assays, the BM-DCs were seeded on IbiTreat channels (BioValley) for 3 h. Adherent cells were pulsed with latex beads (Polysciences) (dilution 1:100) or *Saccharomyces cerevisiae* cells expressing OVA attached to the cell wall (2 × 10^8^/ml) in complete medium for 5 min at 37°C. The free particles were removed by washing with cold PBS, and cells were chased in complete medium. At the end of each chase period, the cells were fixed, permeabilized, and stained.

Immunofluorescence images were acquired with a Leica SP8 confocal microscope using a 40× oil immersion objective. Alternatively and where indicated, images were acquired with a Leica DMI 6000 microscope equipped with a piezoelectric-driven stage and Optophotonics XF100-2 [fluorescein isothiocayanate (FITC)], XF102-2 (Texas Red), and XF06 [4′,6-diamidino-2-phenylindole (DAPI)] filters, and processed for 3D deconvolution using MetamorphTM 6.3.7.

### Image Analysis

Marker colocalization and signal intensity were evaluated using ImageJ. For each image, a stack of at least 10 planes was acquired, and only non-saturated images were measured. A manual threshold was established for each channel before image analysis. Individual cells were delimitated with the freehand selection tool and considered as region of interest (ROI) in ImageJ. For colocalization studies, all images were first translated to a binary image (black pixel intensity = 0; white pixel intensity = 1). The binary images for green and red channels were multiplied to create a mask that encompasses the pixels present in both channels. The areas of green pixels, red pixels, and pixels of the mask were calculated using the plugin “measure stack” of ImageJ. The percentage of green pixels that colocalized with red pixels was calculated as the ratio of the sum of area of pixels in the mask divided by the sum of the area of green pixels.

For measurement of endosomal and Golgi STX6 intensity, a z-projection of the stack image was performed using the sum projection method. On the z-projection image, two individual ROIs were delimitated for each cell analyzed, one for the Golgi stacks and one for the cellular area outside Golgi. Measurements performed with ImageJ were limited to threshold and included ROI area, ROI mean fluorescence intensity, and integrated density. The results were reported as mean of fluorescence or integrated density/area. Statistical analysis was performed with GraphPad Prism software using unpaired *t*-tests.

### Flow Cytometry and Sorting

Bone marrow-derived DCs were incubated on ice with fluorochrome- or biotin-conjugated CD11b, CD11c, and AF6-88.5 antibodies diluted in PBS with 2% FCS. APC/Cy7-streptavidin (Biolegend) was used as a secondary reagent and 7-AAD (5 μl/sample) to discriminate live and dead cells. BD Fortessa^TM^ and fluorescence-activated cell sorting (FACS) ARIA-II^TM^ machines were used for cell analysis and sorting, respectively.

### Immunoblots

Latex bead-containing phagosomes were prepared from BM-DCs by sucrose gradients according to published procedures (Desjardins et al., 1994). Briefly, 1.5 × 10^8^ BM-DCs were fed 0.5 ml deep-blue latex beads (Sigma). Cells were lysed mechanically, excess beads were removed by FCS flotation, and phagosomes were purified *via* a sucrose gradient by ultracentrifugation. Phagosomes were finally lysed in 1% 3-[(3-cholamidopropyl)dimethylammonio]-**1**-propanesulfonate hydrate (CHAPS), the protein concentration was measured with the Bradford reagent (BioRad), and the protein composition was analyzed by immunoblots.

### PhagoFACS

Here, 0.5 mg/ml OVA (Worthington) or ultrapure BSA (Sigma) were covalently coupled to 3 mm latex aminobeads (Biovalley). Bone marrow-derived DCs were pulsed with beads for 10 (Figure 4) or 20 (Figure 2A) min; the excess of non-phagocytized beads was removed by FCS flotation gradients, and the cells were harvested immediately or after another 10 min (Figure 4) or 40 min (Figure 2A) incubation of cells at 37°C. In the experiment shown in Figure 4, half of the BM-DCs were preincubated with 1 μM IRAP inhibitor DG-026A for 2 h and the inhibitor was maintained during phagocytosis. After completion of the time course, the cells were mechanically lysed with a syringe in 250 mM sucrose, 3 mM imidazole, 1 mM dithiothreitol (DTT), and phagosomes were enriched by low-speed centrifugation, fixed in 1% PFA, stained with the lipid marker CellMask (Invitrogen) and Rab7, LAMP1, or OVA antibodies, and analyzed on a Canto II or Gallios cytometer.

### Phagosomal pH

The pH-sensitive dye carboxyfluorescein succinimidyl ester (CFSE) and the pH-insensitive dye AF647 were coupled to latex aminobeads (Polysciences) and added to DCs for phagocytosis. After removal of excess beads, the decrease of the CFSE-emitted fluorescence intensity was measured over time by flow cytometry. The signal was corrected for non-specific dye degradation with the AF647 signal. pH values were obtained by plotting the CFSE mean fluorescence intensity values to a calibration curve that was obtained by lysing phagosomes containing fluorochrome-conjugated beads in 1% Tween-20 solutions of known pH.

### Microbicidal Activity *in vitro*

Bone marrow-derived DCs were incubated with *Pseudomonas aeruginosa* or *S. cerevisiae* for 3 h at a microbe/DC ratio of 1. Then, microbes were washed away and the DCs were lysed in 0.1% Triton. The lysates were plated at different dilutions on yeast and bacterial culture plates, respectively, and colony formation was quantified after 18 h of culture. For *Aspergillus fumigatus* conidia phagocytosis, BM-DCs were incubated with conidia at a conidia/DC ratio of 2, in 24-well plates. The plates were centrifuged at 4°C for 30 min followed by extensive washing with cold PBS to remove free conidia. The number of conidia bound to BM-DCs membrane was identical for wild-type (wt) and IRAP^–/–^ cells. The BM-DCs were incubated for 5 h at 37°C in complete medium. Finally, the cells were lysed in 5 mM EDTA and 0.1% Tween 20 in H_2_O, and the lysates were plated on agar plates. *A. fumigatus* colonies were counted 48 h later.

### Phagosomal Peptide Transport Assay

Three-micron latex beads (Polysciences) were coated by passive adsorption with 100 μg/ml rabbit immunoglobulin and incubated at a ratio of 3:1 for 20 min at 37°C for phagocytosis with BM-DCs. After elimination of free beads by FCS gradient, half of the preparation was disrupted immediately for phagosome preparation and the other half was incubated for another 20 min at 37°C to initiate phagosome maturation. Then, cells were disrupted mechanically in 220 mM sucrose, 3 mM imidazole, 1 mM DTT, and 5 mM MgCl_2_, and phagosomes were pelleted after removal of nuclei. Phagosomes were preincubated during 5 min with 0.9 mM ATP, 0.2 mg/ml creatine kinase, and 400 mM creatine phosphate and incubated for 15 min at 20°C or 4°C with 5 μM peptide RRYNAC(FITC)TEL (R9L-FITC) obtained at ∼80% purity from Pepscan or Sigma-Genosys. Finally, the phagosomes were washed with PBS, stained with 7.5 μg/ml CellMask Deep Red^TM^ membrane stain (Invitrogen) for 30 min at 4°C, and analyzed for peptide accumulation by FACS. Phagosomes incubated at 4°C served as negative controls.

### Cross-Presentation Assays

Here, 2 × 10^6^ BM-DCs were transfected on day 5 of culture with 2 μg of plasmids encoding wt or mutant forms of IRAP or Rab14 using the Amaxa (Lonza) nucleofection kit for mouse immature DCs and program Y-001. On day 7, the cells were stained with CD11b and CD11c antibodies for 20 min, sorted as 7-AAD^–^CD11b^+^CD11c^+^GFP^+^ cells, and seeded at a concentration of 15,000–20,000 cells/well into 96-well round-bottom culture plates. After 1 h, CD8^+^ T cells purified from lymph nodes of OT-I mice were added to the culture for 20 h at a ratio T/BM-DCs: 1.5/1. To assess T cell activation, IL-2 concentration in supernatants was measured by sandwich ELISA using Nunc Maxisorp plates, streptavidin/horseradish peroxidase (Thermo Fisher Scientific), and OptEIA TMB substrate (BD Biosciences). Results represent the means of duplicate wells.

### Peptidase Activity Assays

IRAP^–/–^ mouse epithelial fibroblasts were transfected with wt IRAP or the E465A mutant by electroporation. Two days later, 2 × 10^6^ surviving cells were lysed in 1% Triton, and IRAP was immunoprecipitated with specific mouse antibodies immobilized on Sepharose 4B beads. Half of the beads was resuspended in gel loading buffer and analyzed by immunoblot for IRAP content. The other half was used to prepare triplicate samples and incubated for 15 min with 50 μM Arg-AMC with continuous measuring of the fluorescence signal at 460 nm using a Mithras^TM^ fluorometer (Berthold).

### Cloning of Wild-Type and Mutant IRAP in pIRES2-eGFP

A cDNA coding for IRAP truncated by the 79 aminoterminal amino acids and extended by a carboxyterminal HA tag was amplified from mouse spleen cDNA using the following primers: 5′-ACC GGT GCC ACC ATG CTA CTA GTA AAT CAG TCA C (*Age*I site underlined) and 5′-GAG CTC CTA *GGC GTA GTC GGG CAC GTC GTA GGG GTA* TCC CGA CAG CCA CTG GGA GAG-3′ (HA tag in italics, *Xho*I site underlined). The PCR product was cloned in the pMAX-GFP vector (Lonza) between the *Age*I and *Xho*I sites, thereby replacing the GFP cDNA and resulting in the plasmid pMAX-IRAP. The absence of errors was confirmed by sequencing. A cDNA encoding truncated inactive IRAP was obtained by the megaprimer method. A megaprimer of 235 bp was produced by PCR using pMAX-IRAP as template and the primers: 5′-CT AAA ATC ATT GCT CAC GCA CTG GCA CAT CAG TGG-3′ (mutated base underlined) and 5′-GAA GAC TGA ACA GAT GAT GAT ATT GGA TGA-3′, the latter primer being located downstream of an *Eco*RI site in the IRAP cDNA. A second PCR product was obtained using the megaprimer and the sense primer 5′-ACC GGT GCC ACC ATG CTA CTA GTA AAT CAG TCA C (*Age*I site underlined). This PCR product was used to replace the fragment between the *Age*I and *Eco*RI sites in pMAX-IRAP, creating pMAX-IRAP E465A, followed by sequencing to confirm the mutation. To obtain plasmids encoding full-length IRAP, the first 650 bp of IRAP were amplified using the primers 5′-GCT AGC GCC ACC ATG GAG TCC TTT ACC AAT GA-3′ (*Nhe*I underlined) and 5′-TTG AAA TAT TAT GTC CTG TGC TAT-3′, using as template the cDNA clone MGC_144171 (plasmid IMAGE_40098425, obtained from Open Biosystems) corresponding to Genbank accession number BC120926.1. The N-terminal IRAP fragment was cloned in pCR Blunt (Invitrogen) and sequenced. The C-terminal fragments of wt and inactive IRAP were removed from the pMAX plasmids using *Eco*RV and *Xho*I and cloned together with the N-terminal fragment, excised using *Nhe*I and *Eco*RV, between the *Nhe*I and *Xho*I sites in pCDH-EF1alpha vector (System Biosciences). Finally, the full-length IRAP inserts were transferred from pCDH-EF1alpha into pIRES2-EGFP (Clontech) as *Nhe*I and *Xho*I fragments.

### Lentivirus Production and Infection

The plasmids pLK0.1-puro carrying shRNA sequences specific for Sec22b (TRCN0000115089, also used by Cebrian et al., 2011) and a puromycin resistance gene were purchased from Open Biosystems. The control pLK0.1 plasmid carrying a non-targeting shRNA sequence (SHC002H) was purchased from Sigma Aldrich. The pLK0.1 plasmids were co-transfected with the packaging plasmids pCMVDelta8.2 and the envelope plasmid pMD2G into HEK-293-FT cells *via* calcium chloride transfection. Five hours post-transfection, the buffer was exchanged for complete Dulbecco’s modified Eagle’s medium (DMEM) and virus-containing supernatant was collected 24, 36, and 72 h post-transfection. The supernatants were concentrated in Centricon 70 devices (Millipore). To determine virus titers, NIH3T3 fibroblasts were transduced with several dilutions of viral supernatants. Two days later, cells were harvested, genomic DNA was isolated, and the viral gene *fugw* was amplified by qPCR, and copy numbers were determined by plotting the obtained signal against dilutions of a *fugw*-encoding plasmid amplified in the same experiment.

Red blood cell-depleted bone marrow cells on day 3 of culture in the presence of GM-CSF were put in contact with lentiviral supernatants at a multiplicity of infection (MOI) of 10 supplemented with 8 μg/ml polybrene and centrifuged for 100 min at 32°C. Subsequently, supernatants were washed off and replaced by BM-DC culture medium. Non-transduced cells were depleted by addition of 5 μg/ml puromycin on day 5. Transduced cells were used on days 7–8 of culture. The efficiency of knockdown was confirmed by immunoblot and qPCR.

### Statistical Analysis

Statistical analyses including regression analysis, unpaired *t*-tests, and two-way ANOVA with *p*-values adjusted by Dunnett correction test (cross-presentation) were performed using GraphPad Prism^TM^ software (version 7.0). For cross-presentation experiments, the mean values of four independent experiments were pooled and statistical significance was determined for every dose of antigen (parameter 1) and for every cell treatment (parameter 2) by comparing both the vector-IRAP^+/+^ and the IRAP_E__465__A_-IRAP^–/–^ conditions to the vector-IRAP^–/–^ condition. For visual simplicity, the graph shows the p value applying to the three higher antigen concentrations.

## Results

### Loss of Insulin-Regulated Aminopeptidase Accelerates Phagosome Maturation

**FIGURE 1:**
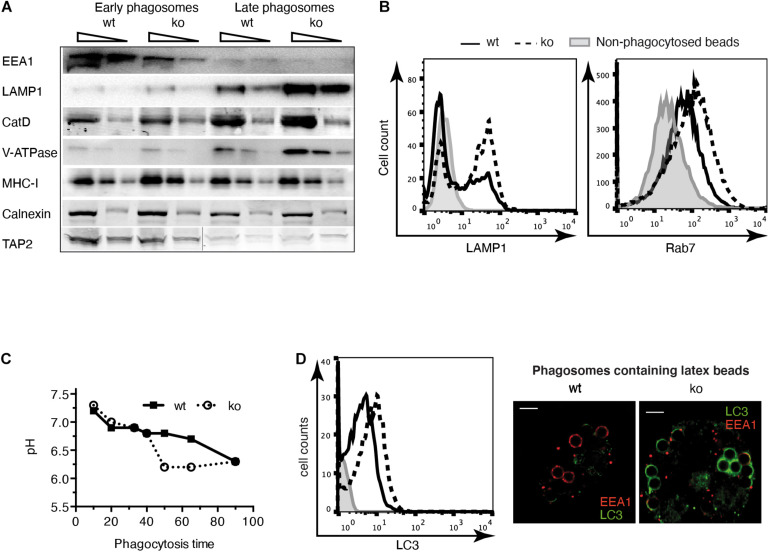
Phagosome maturation is accelerated in insulin-regulated aminopeptidase (IRAP) ^– /–^ dendritic cells (DCs). **(A)** Latex bead phagosomes were isolated from wild-type (wt) or IRAP^– /–^ bone marrow-derived DCs (BM-DCs) by sucrose gradients after a 20-min pulse (early) and after 120 min of chase (late). Equal protein amounts were analyzed by immunoblot for the markers indicated. One of three or more experiments. CatD, cathepsin D. **(B)** BM-DCs were pulsed for 20 min with latex beads and mechanically disrupted. Phagosomes in the post-nuclear supernatant were fixed, identified with the lipid marker Cellmask^TM^, stained for lysosome-associated membrane protein (LAMP)1 and Rab7, and analyzed by flow cytometry. One out of 10 (LAMP1) and three (Rab7) experiments. **(C)** The fluorescence intensity of phagocytosed latex beads conjugated to the pH-sensitive dye carboxyfluorescein succinimidyl ester (CFSE) and the pH-insensitive dye AF647 was monitored over time by flow cytometry. The ratio of CFSE to AF647 fluorescence was converted to pH values using a standard curve of beads in solutions of known pH. One of three similar experiments. **(D)** BM-DCs were pulsed for 20 min with latex beads, and LC3 staining of phagosomes containing single beads was performed as described in panel **(B)**. The images to the right show BM-DCs pulsed for 5 min with latex beads, chased for 15 min, and stained for LC3 and EEA1.

Aiming to identify the peptidases involved in trimming the amino-terminus of MHC-I-presented antigenic peptides, we have previously reported that IRAP is rapidly recruited to newly formed phagosomes (Saveanu et al., 2009; Weimershaus et al., 2012, 2018). Here, comparing wt with IRAP^–/–^ DCs lacking Rab14^+^ storage endosomes, we set out to investigate in detail how this endosome species modulates the dynamic changes and molecular properties of phagosomes in the course of their maturation process. To this aim, we isolated latex-bead phagosomes from wt and IRAP^–/–^ DCs and analyzed their content by immunoblot (Figure 1A). While neither the recruitment of the ER proteins Calnexin and TAP nor the amount of MHC-I molecules in phagosomes depended on the expression of IRAP, phagosomes from IRAP^–/–^ DCs prematurely lost the early endosomal antigen (EEA) 1 and acquired more rapidly late endosomal and lysosomal markers such as cathepsin (Cat)D, the proton pump V-ATPase, and LAMP-1 (Figure 1A). To confirm these results by an alternative approach, we performed phagoFACS experiments, which allow for detection of selected membrane markers on bead-containing phagosomes at a given time point of maturation *via* flow cytometry (Savina et al., 2010). In agreement with the results from the immunoblot analysis, FACS staining for the late endosomal/lysosomal markers LAMP-1 and Rab7 showed higher fluorescence intensities on phagosomes isolated from IRAP^–/–^ DCs, indicating an accelerated phagosome maturation in these cells compared to wt DCs (Figure 1B). These changes in the presence of endosomal markers on the phagosomal membrane were accompanied by a stronger acidification measured in IRAP^–/–^ phagosomes (Figure 1C).

Considering the published role of LAP in enhancing phagosome maturation, we wondered if IRAP^–/–^ phagosomes displayed more LC3 on their membranes. Using phagoFACS and confocal imaging, we observed that early phagosomes from cells lacking IRAP showed stronger LC3 staining (Figure 1D). These results indicate that IRAP endosomes contribute to regulating phagosome maturation, stabilizing early endosomal markers and presumably a higher pH while limiting recruitment of a key element of the highly degradative LAP pathway.

**FIGURE 2:**
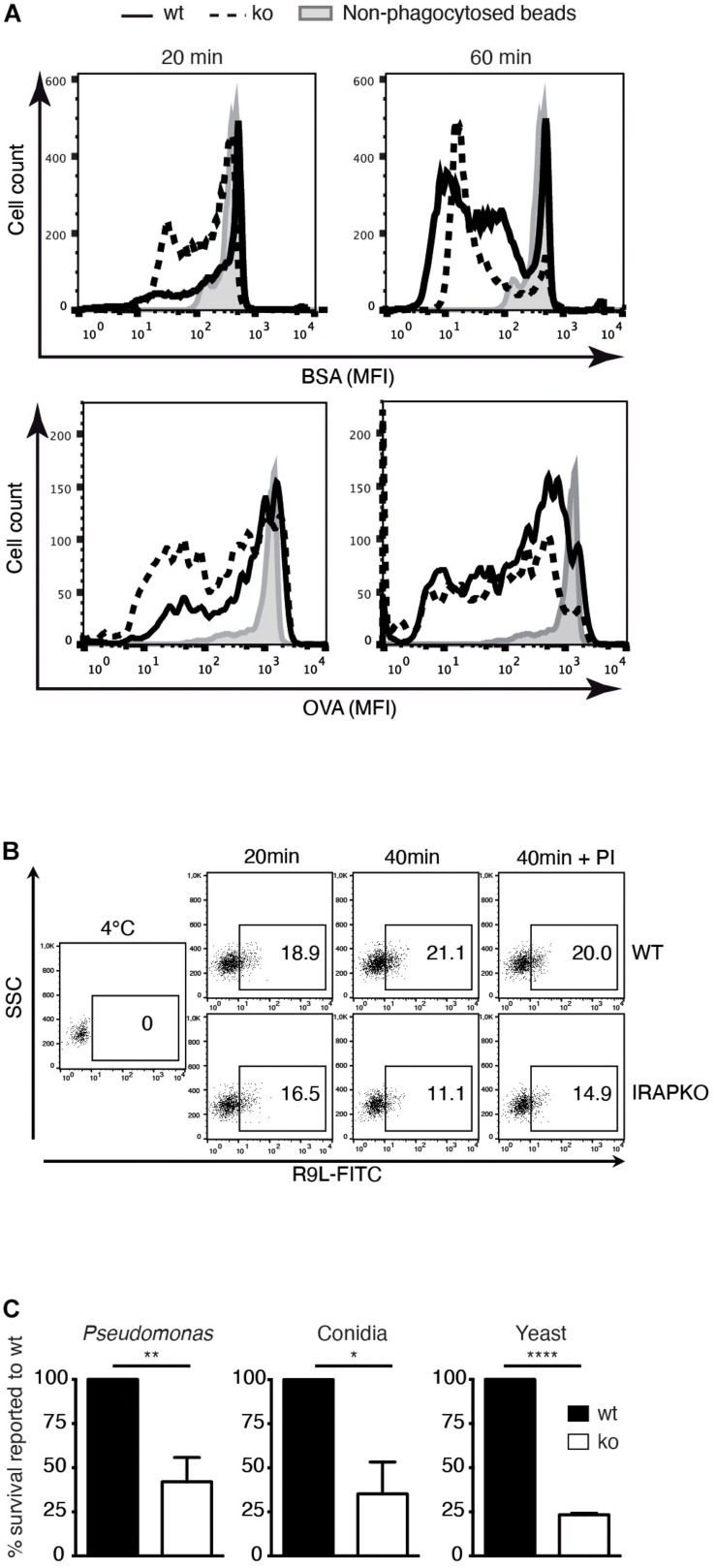
Insulin-regulated aminopeptidase (IRAP) ^– /–^ phagosomes are more degradative and microbicidal. **(A)** Wild-type (wt) and IRAP^– /–^ bone marrow-derived dendritic cells (BM-DCs) were pulsed for 20 min with latex beads coupled to ovalbumin (OVA) or bovine serum albumin (BSA) and half of the cells were chased for 40 min. The cells were lysed, and the beads were stained for BSA or OVA and analyzed by flow cytometry. One out of three or more experiments. **(B)** BM-DCs were incubated for 20 min with latex beads, washed, and lysed mechanically immediately or after another 20 min incubation at 37°C for phagosome preparation. The phagosomes obtained from wt and IRAP^– /–^ BM-DCs were incubated for 15 min at 4°C or 20°C with peptide R9L-FITC followed by analysis of peptide accumulation by fluorescence-activated cell sorting (FACS). One representative of three experiments is shown. **(C)** Wt and IRAP^– /–^ BM-DCs were allowed to phagocytose the indicated microbes and incubated for 3 h (*Pseudomonas*), 5 h (*Aspergillus fumigatus* conidia), or 6 h (*Saccharomyces cerevisiae*). The microbe survival was determined as the number of colony-forming units (cfu) in solid cultures. The numbers represent the means ± SEM of three independent experiments and are expressed as percent of survival in wt cells. *p < 0.02, **p < 0.002, ***p < 0.0001.

As accelerated phagosome maturation and lower pH are generally associated with increased substrate degradation, we next analyzed the proteolysis of two model substrates in phagosomes from IRAP^–/–^ and wt DCs. Using OVA- and BSA-coated latex beads, respectively, we observed accelerated protein degradation in IRAP^–/–^ phagosomes (Figure 2A). Similar results were obtained when monitoring accumulation of a reporter peptide in an *in vitro* assay of peptide transport into phagosomes: while peptide accumulated during 40 min in wt phagosomes, with greater amounts in later vesicles, peptide amounts decreased over time in IRAP^–/–^ phagosomes. This was due to the more degradative milieu rather than diminished transport activity in IRAP^–/–^ phagosomes, as addition of protease inhibitors increased the peptide amounts accumulating in the vesicles lacking IRAP, whereas it was without effect in IRAP^+/+^ phagosomes (Figure 2B).

One physiological function of phagosome maturation is the inactivation and killing of potentially harmful content including microbes. We thus compared the intracellular survival of bacteria, fungi, and yeast upon phagocytosis by IRAP^–/–^ and wt DCs. All three microbe species showed significantly diminished survival in IRAP^–/–^ phagosomes, consistent with a more proteolytic and microbicidal content (Figure 2C).

### Insulin-Regulated Aminopeptidase Peptidase Activity Is Required for Antigen Presentation

We have previously shown that IRAP exerts trimming activity toward the amino-terminus of peptides in the MHC-I cross-presentation pathway. IRAP^–/–^ DCs fail to efficiently activate CD8^+^ T cells to cross-presented antigens (Saveanu et al., 2009). However, as accelerated phagosome maturation alone might be sufficient to hamper cross-presentation through increased destruction of antigenic peptides in the phagosome, we sought to dissect the contribution of IRAP peptidase activity to efficient cross-presentation as opposed to a merely structural, i.e., non-enzymatic role of IRAP in storage endosomes and their trafficking.

**FIGURE 3:**
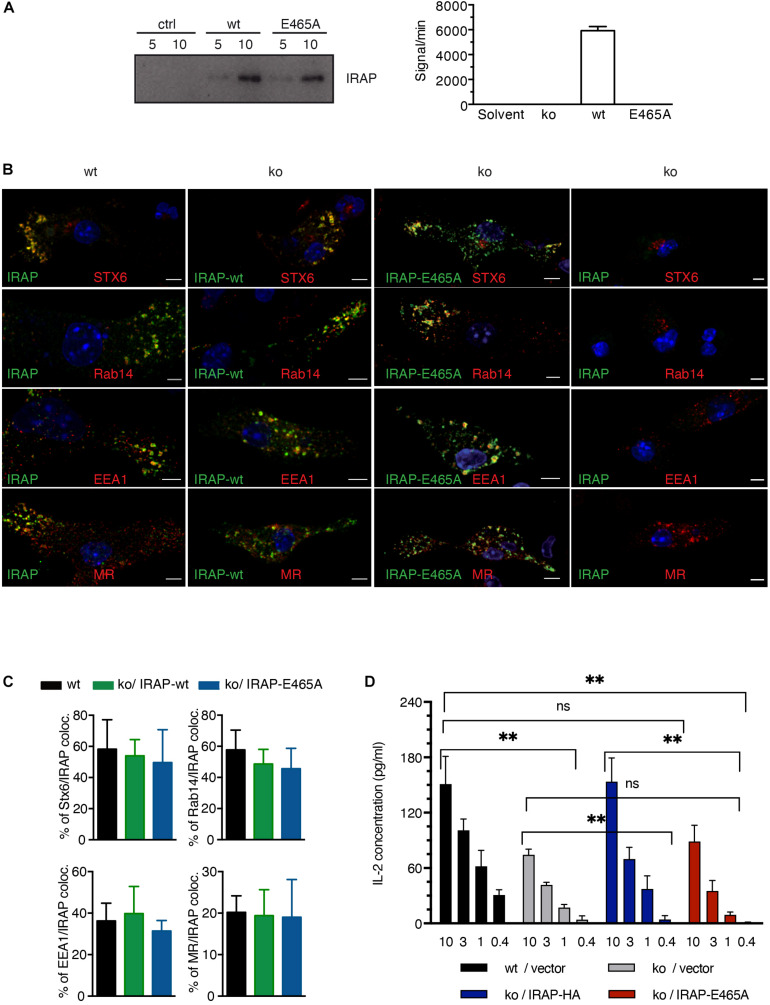
Insulin-regulated aminopeptidase (IRAP) peptidase activity is required for efficient cross-presentation. **(A)** IRAP^–/–^ mouse epithelial fibroblasts (MEFs) were transfected with full-length wild-type (wt) or mutant IRAP or mock-transfected (ctrl), followed by immunoprecipitation of IRAP 2 days later. The precipitates were analyzed for IRAP content by immunoblot and for peptidase activity toward the fluorogenic Arg-AMC substrate using mock-transfected cells as control. **(B)** IRAP^–/–^ bone marrow-derived DCs (BM-DCs) were nucleofected with the plasmid pMAX-IRAP-E465A and analyzed 36 h later by confocal microscopy. Wt and untransfected IRAP^–/–^ BM-DCs are shown as controls. **(C)** The colocalization of storage endosome markers in IRAP wt and IRAP^–/–^ BM-DCs transfected with wt or protease-dead IRAP was analyzed for five representative cells. The graph shows mean co-localization ± SEM. **(D)** IRAP^+/+^ or IRAP^–/–^ BM-DCs were nucleofected with empty pIRES2-GFP or the active site IRAP mutant E465A cloned in pIRES2-GFP. Live transfectants were sorted as GFP^+^7AAD^–^CD11c^+^CD11b^+^ cells and put in contact with graded amounts (mg/ml) of pre-formed complexes formed between MR antibodies and OVA fusion protein and with OT-I T cells. T cell stimulation was assessed by interleukin (IL)-2 ELISA. Histograms show the mean concentration of IL-2 ± SEM after subtraction of values obtained with control Glutamic acid decarboxylase (GAD) fusion protein from four independent experiments. ^∗∗^*p* < 0.01; ns, not significant.

To this end, we generated a peptidase-dead IRAP variant carrying a single amino acid mutation in the catalytic site (E465A), which we overexpressed in IRAP^–/–^ DCs (Figure 3A, upper panel). As expected, in contrast to wt IRAP, the immunoprecipitated peptidase-dead mutant lacked hydrolytic activity toward the fluorogenic substrate Leu-AMC (Figure 3A). While peripheral endosomes staining for Stx6 and Rab14 were essentially absent in IRAP^–/–^ cells, the expression of the peptidase-dead mutant was sufficient to promote the appearance of peripheral endosomes staining for the storage endosome hallmark proteins Stx6, Rab14, EEA1, and mannose receptor (MR; Figure 3B). Wt and mutant IRAP colocalized to the same extent with the endosome markers analyzed, displaying the characteristic strong co-localization with Stx6 and Rab14 and weaker co-staining with EEA1 and MR (Figure 3C). However, while wt IRAP fully restored cross-presentation, the peptidase-dead mutant failed to rescue the cross-presentation defect observed in IRAP^–/–^ DCs (Figure 3D), consistent with a requirement of peptide trimming by IRAP for efficient CD8^+^ T cell priming.

**FIGURE 4:**
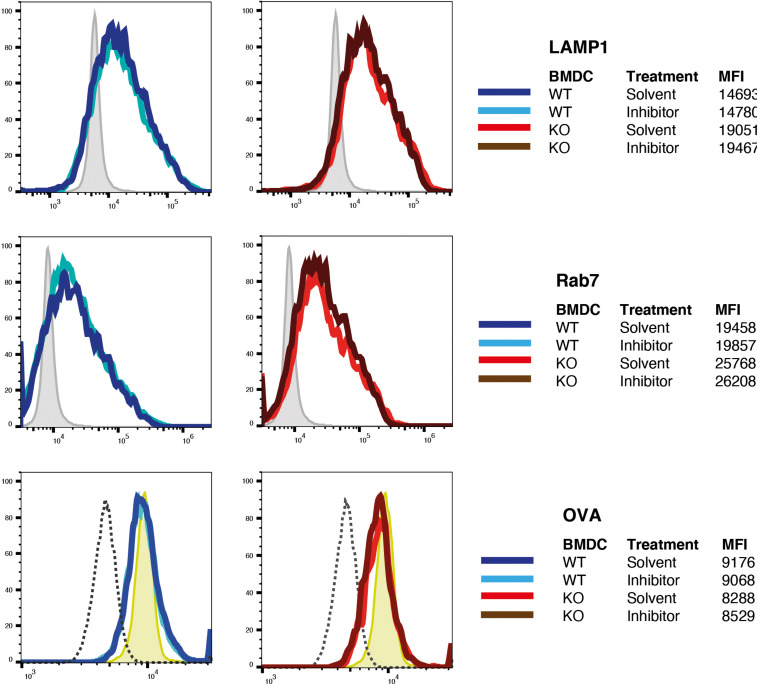
Inhibition of insulin-regulated aminopeptidase (IRAP) protease activity does not alter phagosome maturation. Latex beads covalently coupled to ovalbumin (OVA) were fed to bone marrow-derived dendritic cells (BM-DCs) preincubated with the IRAP inhibitor DG-026A or solvent were incubated for 10 min with OVA-coated latex beads, washed, incubated in the presence of DG-026A for another 10 min at 37°C, and then lysed mechanically for preparation of phagosomes. Fixed phagosomes were stained for the late endosome markers Lamp1 and Rab7 and for OVA and analyzed by flow cytometry. One out of three experiments each performed in duplicate is shown. MFI, mean fluorescence intensity.

However, it was still possible that protease-dead IRAP was sufficient to reconstitute storage endosomes but not normal, i.e., DC-typical attenuated phagosome maturation. Given the poor efficiency of full-length IRAP expression through transfection of BM-DCs, we were not able to obtain sufficient numbers of transfected BM-DCs to perform immunoblot and phagoFACS experiments for monitoring phagosome maturation. However, we reasoned that pharmacological IRAP inhibition should also reveal a potential requirement of proteolysis for physiologic phagosome maturation. We have previously shown that the inhibitor DG-026A reduces cross-presentation by wt BM-DCs to levels observed in IRAP^–/–^ DCs but has no effect on presentation in the latter, indicating selective inhibition of IRAP enzymatic activity in live cells (Kokkala et al., 2016). As expected, early phagosomes prepared from IRAP^–/–^ BM-DCs acquired higher levels of Lamp1 and Rab7 and contained somewhat lower levels of OVA than wt vesicles (Figure 4). However, incubation with DG-026A was without effect on acquisition of late endosome markers and OVA degradation not only in IRAP^–/–^ phagosomes, as expected, but also in wt vesicles. Therefore, we were unable to find experimental evidence for a role of IRAP enzymatic activity in phagosome maturation.

### Insulin-Regulated Aminopeptidase Endosome Stability Depends on Sec22b

**FIGURE 5:**
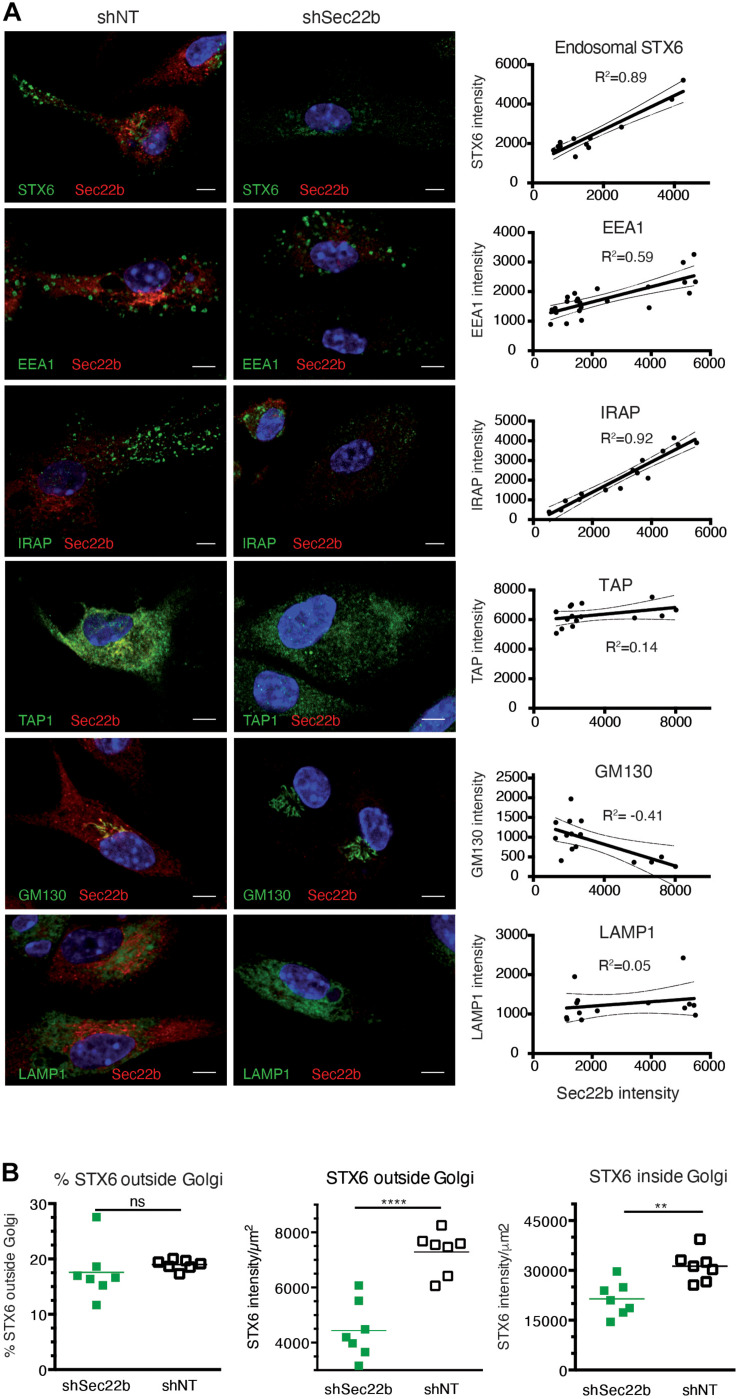
Sec22b knockdown results in severe perturbation of insulin-regulated aminopeptidase (IRAP)^+^STX6^+^ endosomes. **(A)** Bone marrow-derived dendritic cells (BM-DCs) were transduced with shNT or shSec22b lentiviruses, stained for Sec22b, and analyzed by confocal microscopy with identical settings for shNT and shSec22b samples. The adjacent graphs show the correlation between the mean fluorescence intensities for Sec22b and for a second marker, each dot representing a different cell. Correlation coefficients were computed using exclusively unsaturated images. **(B)** BM-DCs were transduced as in panel **(A)** and stained for Sec22b and STX6. The integrated density of STX6 staining was measured in the Golgi area and outside of it for each cell analyzed using unsaturated images. ****p < 0.0001; **p < 0.001; ns, not significant.

Efficient cross-presentation has been suggested to depend on the partial fusion of ER membranes with endosomal compartments in a process relying on the ERGIC SNARE protein Sec22b and its phagosomal fusion partner Stx4 (Cebrian et al., 2011). In order to analyze the importance of Sec22b-mediated membrane fusion in phagosome maturation in IRAP^–/–^ and wt DCs, we knocked down Sec22b expression using a lentivirus-expressed sequence also used by Cebrian and associates. To our surprise, abolishing Sec22b expression profoundly altered the organization of early endosomal structures. Specifically, immunofluorescence imaging revealed a near complete absence of peripheral endosomes staining for Stx6 or IRAP, while EEA1 staining was also slightly affected by Sec22b knockdown in BM-DCs (Figure 5A). Examining the correlation between the endosomal staining intensity for these markers and the efficiency of Sec22b knockdown in individual cells, we observed a strong correlation between Sec22b expression and the presence of IRAP^+^ and Stx6^+^ endosomes, contrasting with a much weaker correlation for EEA1^+^ endosomes (Figure 5A, right panels). TAP and LAMP staining was not affected by Sec22b knockdown. GM130 staining, in contrast, was inversely correlated with Sec22b expression, i.e., increased upon Sec22b deletion. As Stx6 is involved in vesicle traffic between the trans Golgi network (TGN) and endosomes, we asked whether its depletion was selective to endosomes. However, although Sec22b knockdown reduced Stx6 staining outside the Golgi more strongly than within, its distribution was not significantly altered (Figure 5B).

## Discussion

The regulation of phagosomal maturation by myeloid cells is intimately linked to their specific role in the immune system. While macrophages excel in the intracellular destruction of phagocytosed cargo by rapid and efficient phagosome–lysosome fusion, it is widely accepted that the “mild” endosomal milieu in DCs promotes their capacity to cross-present. In this context, we have identified a cellular mechanism of regulated trafficking and fusion of Rab14^+^IRAP^+^ storage endosomes with phagosomes in response to TLR4 engagement (Weimershaus et al., 2018). Here, we further characterize the trafficking dynamics during IRAP endosome-dependent phagosome maturation and report suggestive evidence relating it to previously described mechanisms regulating phagosome maturation such as LAP and the interaction of the ERGIC with post-Golgi vesicles.

We find that the absence of IRAP accelerates the loss of early and the acquisition of late endosomal markers. Consistent with this changed kinetics, IRAP^–/–^ phagosomes display a lower luminal pH and an increased hydrolytic activity, which translates into increased killing of phagocytosed yeast, fungi, and bacteria, as well as increased TLR9 signaling (Babdor et al., 2017). Accelerated phagosome maturation most likely implicates the regulated movement of storage endosomes along microtubules: IRAP’s N-terminus protrudes from the vesicular membrane to the cytosol where it physically interacts with the formin FHOD4, which anchors the endosomes to the actin cytoskeleton (Babdor et al., 2017). Additionally, we have previously shown that IRAP interacts with Tbc1d4/AS160, the Rab14 GAP in DCs (Weimershaus et al., 2018). Tbc1d4/AS160 activity is negatively regulated by the class I phosphoinositide 3-kinase (PI3K)/Akt signaling (Sadacca et al., 2013), a pathway known to be triggered by innate immune receptors and Fc receptors (Nair et al., 2011). Akt enzymatic activity leads to phosphorylation of Tbc1d4/AS160 and to guanosine diphosphate (GDP)–GTP exchange at Rab14. GTP-bound Rab14 associates with the kinesin Kif16b and promotes anterograde transport of IRAP endosomes to the cell periphery. The presence of IRAP and Rab14 in early phagosomes suggests that in DCs, phagocytosis is rapidly followed by fusion with storage endosomes, such that interaction of the two proteins with the cytoskeleton takes control of phagosome maturation (Saveanu et al., 2009; Weimershaus et al., 2012, 2018).

According to our findings in this and our previous report, the loss of IRAP and its static anchoring to the cytoskeleton is sufficient to promote destabilization of storage endosomes and at the same time abolish plus-end-directed vesicle transport *via* the regulated Rab14-Kif16b hub, such that transport toward and fusion with lysosomes become dominant. In addition, *via* its recently discovered interaction with the retromer complex (Pan et al., 2017), IRAP might contribute to the retrieval of specific subdomains and cargos from early endosomes and phagosomes to trans-Golgi-derived vesicles, avoiding thus their targeting to lysosomes, a possibility that needs further investigation.

Interestingly, IRAP^–/–^ phagosomes acquired more lipidated LC3 on their membranes. LC3-associated phagocytosis had first been described in murine macrophages as a non-canonical function of autophagy proteins (Sanjuan et al., 2007), resulting in accelerated phagosome maturation compared to phagocytosis without LC3 implication. Several membrane receptors have been described to trigger LAP, including TLR1/2/6, TLR4, TIM4, and FcR (Martinez, 2018). The initial signaling cascade, however, triggering the recruitment of the class III PI3K complex upstream of LC3 association, remains elusive, as well as the mechanisms through which the autophagy machinery promotes phagosome–lysosome fusion. Nevertheless, our findings suggest that the factors delivered to DC phagosomes by TLR4 or FcR-triggered fusion with IRAP storage endosomes “override” early signaling events leading to LC3 association with phagosomes because efficient LC3 association is only observed in IRAP^–/–^ DCs. Analyzing the presence of Rubicon (a LAP-exclusive class III PI3K subunit) or the resulting phosphoinositide-3-phosphate levels on nascent phagosomes in IRAP^–/–^ cells could provide further insight into the early membrane-modifying events that are regulated by IRAP.

The analysis of purified phagosomes did not reveal differences in the transport of MHC-I molecules to this compartment between wt and IRAP^–/–^ cells. The exact relationship, however, between IRAP^+^ storage endosomes and the DC endocytic recycling compartment (ERC) described by Nair-Gupta et al. (2014) remains unclear. This compartment is defined by VAMP3/VAMP8/Rab11 and harbors stocks of MHC-I molecules delivered upon TLR4 signaling for peptide loading to phagosomes. Note that despite the suggestive name of the ERC, the origin of its MHC-I molecules is unclear, as we have been unable to observe delivery of internalized recycling MHC-I molecules to it (Montealegre et al., 2019). IRAP deficiency did not affect the recruitment of ER-derived components (Calnexin, TAP) to phagosomes, which positions storage endosomes offside the trafficking and fusion events of ER membranes with phagosomes.

Interestingly, we observed that the knockdown of the ERGIC-SNARE Sec22b destabilized IRAP^+^ storage endosomes in the steady-state. A role of this SNARE in intracellular IRAP trafficking has not been described; however, indirect evidence from studies on GSVs in insulin-responsive cells might help to explain our observations: in the absence of insulin signaling, GSVs are retained at a pre-Golgi location by the action of the Golgi tether proteins TUG, Golgin-160, and p115, the latter of which directly binds IRAP (Hosaka et al., 2005; Williams et al., 2006). TUG, Golgin-160, and p115 localize to the cis-Golgi, ERGIC, and ER exit sites, where p115 has been shown to form stable complexes with Sec22b (Wang et al., 2015). Compromised formation of Sec22b-p115 complexes might result in misdirection of IRAP from the ERGIC or in its retrieval to the ER for eventual degradation; given the essential absence of IRAP in Sec22b knockdown BM-DCs, we favor the latter possibility. Whatever the mechanism underlying IRAP depletion upon Sec22b knockdown, our observation suggests a potential explanation for the previously unexplained accelerating effect of Sec22b knockdown on phagosome maturation (Cebrian et al., 2011). Next to affecting the delivery of ER membrane components to phagosomes, Sec22b may be required for correct formation of storage endosomes, which in turn regulate phagosome maturation

Finally, we address the question of the dual role of IRAP as a trimming aminopeptidase and a constitutive component of storage endosomes in cross-presentation. We show that protease-dead IRAP fully reconstitutes IRAP^+^ endosomes with a typical set of markers and that an IRAP inhibitor does not accelerate phenocopy IRAP knockout, i.e., does not accelerate phagosome maturation. Taken together, these findings suggest that IRAP has a structural role in storage endosomes and that this role is sufficient to mediate normal phagosome maturation. As DC-typical phagosome maturation has a well-documented importance in cross-presentation, it is plausible and likely that the structural role of IRAP has an independent effect on cross-presentation. Conversely, the fact that cross-presentation by BM-DCs expressing protease-dead IRAP is indistinguishable from cross-presentation by IRAP^–/–^ cells lends credibility to a requirement of peptide trimming by IRAP for cross-presentation.

Interestingly, active proteasome complexes have recently been detected in endocytic DC vesicles, the precise identity of which remains to be determined (Sengupta et al., 2019). In our hands, cross-presentation of soluble, receptor-targeted, or particle-associated OVA is proteasome-dependent. Concerning TAP dependence, we have previously shown that the detrimental effect of TAP deficiency on cross-presentation of phagocytosed (but not receptor-targeted soluble) OVA by BM-DCs can be overcome by restoring cell surface class I levels through low-temperature incubation (Merzougui et al., 2011). In other words, particulate OVA cross-presentation by BM-DCs requires antigen degradation by proteasome complexes but not antigenic peptide transport by TAP, features shared by the experimental system employed by Sengupta et al. (2019). It is therefore conceivable that proteasome complexes reside in IRAP^+^ vesicles, which then would contain a complete proteolytic chain for production of antigenic peptides. As the selectivity of IRAP trimming overlaps significantly with that of ER(A)P (Georgiadou et al., 2010; Mpakali et al., 2015), such a scenario would increase the likelihood that similar peptides are produced and presented in the endogenous and exogenous antigen presentation pathways, ensuring efficient recognition of tissue cells by CD8^+^ T cells primed by cross-presenting DCs.

Collectively, our findings highlight the important and dual role of IRAP^+^Rab14^+^ endosomes in optimizing cross-presentation and suggest that their availability may physiologically contribute to regulating the capacity of DCs to cross-prime CD8^+^ T cell responses.

## Data Availability Statement

The raw data supporting the conclusions of this article will be made available by the authors, without undue reservation.

## Ethics Statement

The animal study was reviewed and approved by Comité d’Éthique pour l’Expérimentation Animale Paris Descartes.

## Author Contributions

MW, F-XM, and LS designed, performed, and interpreted the experiments and co-wrote the manuscript. ML and IE performed the experiments. PE designed and interpreted the research and co-wrote the manuscript. All authors contributed to the article and approved the submitted version.

## Conflict of Interest

The authors declare that the research was conducted in the absence of any commercial or financial relationships that could be construed as a potential conflict of interest.
